# Effective Antimicrobial Prophylaxis in Surgery: The Relevance and Role of Pharmacokinetics-Pharmacodynamics

**DOI:** 10.3390/antibiotics12121738

**Published:** 2023-12-14

**Authors:** Sheryl A. Zelenitsky

**Affiliations:** 1 College of Pharmacy, Rady Faculty of Health Sciences, University of Manitoba, Winnipeg, MB R3E 0T5, Canada; zelenits@umanitoba.ca; 2 Department of Pharmacy, St. Boniface Hospital, Winnipeg, MB R2H 2A6, Canada

**Keywords:** antibiotic prophylaxis, surgical prophylaxis, cefazolin, pharmacokinetics, pharmacodynamics, surgical site infection

## Abstract

Appropriate surgical antimicrobial prophylaxis (SAP) is an important measure in preventing surgical site infections (SSIs). Although antimicrobial pharmacokinetics–pharmacodynamics (PKPD) is integral to optimizing antibiotic dosing for the treatment of infections, there is less research on preventing infections postsurgery. Whereas clinical studies of SAP dose, preincision timing, and redosing are informative, it is difficult to isolate their effect on SSI outcomes. Antimicrobial PKPD aims to explain the complex relationship between antibiotic exposure during surgery and the subsequent development of SSI. It accounts for the many factors that influence the PKs and antibiotic concentrations in patients and considers the susceptibilities of bacteria most likely to contaminate the surgical site. This narrative review examines the relevance and role of PKPD in providing effective SAP. The dose–response relationship i.e., association between lower dose and SSI in cefazolin prophylaxis is discussed. A comprehensive review of the evidence for an antibiotic concentration–response (SSI) relationship in SAP is also presented. Finally, PKPD considerations for improving SAP are explored with a focus on cefazolin prophylaxis in adults and outstanding questions regarding its dose, preincision timing, and redosing during surgery.

## 1. Introduction

Surgical site infections (SSIs) are significant complications that increase patient morbidity, mortality, and healthcare costs [1,2]. In a meta-analysis of nearly half a million patients worldwide, the pooled cumulative incidence of SSI in general surgery was 11% [3]. Although the risk is variable among patients, types of surgery, and surgical settings, it is estimated that over half of infections can be prevented by measures including appropriate surgical antimicrobial prophylaxis (SAP) [4,5]. SAP involves the use of systemic antibiotics during surgery to eradicate bacteria from the surgical wound, thereby preventing contamination and reducing the risk of infection [6]. The most suitable antibiotics are bactericidal, cover the most likely SSI pathogens, have minimal impact on antimicrobial resistance [7], have a favorable safety profile, and are cost effective [8]. First or second generation cephalosporins, particularly cefazolin, are the most used and studied antibiotics for surgical prophylaxis.

Antibiotic dosing for the treatment of infections is based on antimicrobial pharmacokinetics–pharmacodynamics (PKPD). Whereas PKs characterize the antibiotic concentrations achieved in the patient through the processes of absorption, distribution, metabolism, and excretion, PDs describe the antimicrobial effects on the pathogen. Combined, PKPD explains the complex relationship between antibiotic exposure, microbiological response, and clinical outcomes [9,10]. PKPD modelling is used to determine dosing for new agents [11,12,13] and revise dosing to conserve the effectiveness of older antibiotics [14,15,16]. It is particularly important in the study of dosing for special populations with altered PKs, e.g., neonates, obese, renal replacement, critically ill [17]. PKPD is also used to translate in vitro antimicrobial susceptibility testing to clinical practice by identifying meaningful MIC (minimum inhibitory concentration) breakpoints that categorize isolates as susceptible or resistant to specific antibiotic therapies [18,19].

Although there is less PKPD research on preventing infections, there is broad consensus that SAP should provide adequate coverage throughout surgery, from incision to closure [8,20,21,22]. The PKs of antibiotics are variable in surgical patients due to heterogeneity in age, body weight, renal function, and comorbidities. Furthermore, the PDs apply to bacteria most likely to contaminate the surgical site as opposed to an infecting pathogen. PKPD investigations relating antibiotic exposure during surgery to the subsequent development of SSI remain limited. Even so, Monte Carlo simulations are used to evaluate SAP regimens by incorporating PKPD principles and predicting target attainment in simulated surgical populations.

This narrative review examines the relevance and role of PKPD in providing effective SAP. The dose–response relationship i.e., association between lower dose and SSI in cefazolin prophylaxis is discussed. A comprehensive review of the evidence for an antibiotic concentration–response (SSI) relationship in SAP is also presented. Finally, PKPD considerations for improving SAP are explored with a focus on cefazolin prophylaxis in adults and outstanding questions regarding its dose, preincision timing, and redosing during surgery.

## 2. Dose–Response Relationship in Cefazolin Prophylaxis

Examples of clinical studies that characterize the association between lower cefazolin dose and an increased risk of SSI are detailed in Table 1. Such investigations are usually retrospective or observational studies that find higher rates of infection when cefazolin is underdosed according to practice guidelines, e.g., 2 g for patients <120 kg or 3 g for those ≥120 kg, administered within 1 h prior to incision [8]. Given the use of standard cefazolin doses over a wide range of body weight, an association between SSI and lower calculated mg/kg doses received by patients may also be observed.

Although clinical studies relating the SAP dose to SSI are informative, there are significant limitations that can impede the detection of a dose–SSI relationship. First, without the PKs, dose is a poor predictor of the antibiotic concentrations achieved in patients and antimicrobial activity in vivo. Furthermore, dose is only one component of the SAP regimen which also relies on appropriate preincision timing and redosing during surgery. Finally, SSI outcomes are affected by many other patient- and surgery-related factors such as obesity, diabetes, immune status, type of surgery, wound classification, and surgical duration. Heavier body weight (i.e., obesity) is a significant risk factor [23,24] which often complicates the study of cefazolin dosing for surgical prophylaxis. With the use of standard doses, there is a negative correlation between body weight and the mg/kg dose received by patients. For example, the mg/kg dose delivered by 2 g of cefazolin decreases as body weight increases to the cut-off of 120 kg, thereby confounding the effects of heavier body weight and lower dose as risk factors for SSI.

In a retrospective, case–control study, Cies et al. investigated whether body mass index (BMI) affected infection rates in pediatric orthopedic surgery [25]. The mean age of patients was 12.8 years, ranging from 3 years to 9 years. Overall, the American Society of Anesthesiologists (ASA) class II or III (*p* = 0.006) and surgery >2 h (OR = 2.45, *p* = 0.01) were independently associated with SSI. The study also included a subgroup analysis of cefazolin prophylaxis stratified by dosing practice, i.e., standard-dose of 1 g for patients ≥70 kg and weight-based dose of 20–30 mg/kg to a maximum of 1 g for those <70 kg. The infection rate was significantly higher in patients who received the standard compared to weight-based dosing (35.9% versus 20.5%, *p* = 0.045). The authors noted that the groups were also stratified by body weight, but observed that overall patients who were underweight, not overweight, were more likely to develop SSI than those who were a healthy weight (*p* = 0.023). They concluded that weight-based dosing of cefazolin was appropriate for their patient population, but dosing to a maximum of 1 g increased the risk of infection.

Abdel Jalil et al. conducted a prospective, observational study of the frequency and potential risk factors for SSI in caesarean surgery [26]. Compliance with a new institutional guideline to use 2 g instead of 1 g of cefazolin preoperatively for patients <120 kg was also evaluated. A dose–response relationship was observed where patients who developed SSI received a lower calculated mg/kg dose than those without infection (18.7 mg/kg versus 22.2 mg/kg, *p* = 0.037). Furthermore, a lower mg/kg dose of cefazolin independently associated with SSI (*p* = 0.035) in multivariable logistic regression analysis, as were longer hospital stay (*p* = 0.001), higher BMI (*p* = 0.003), and delivery beyond the 40th week (*p* = 0.005). As expected, the mg/kg dose was negatively correlated with BMI. The investigators concluded that using 2 g versus 1 g of cefazolin was beneficial, predicting that it would reduce the risk of infection by 36% in their typical patient undergoing caesarean surgery.

Rondon et al. reviewed 17,393 cases of total joint arthroplasty at a single center over 13 years to determine what proportion of cefazolin prophylaxis was adequately dosed and whether underdosing was associated with prosthetic joint infection (PJI) [27]. Underdosing was defined as <1 g for any patient, <2 g for those between 60 kg and 120 kg, or <3 g for those ≥120 kg. The preoperative dose was given within 1 h in all cases. Cefazolin was adequately dosed, underdosed, and overdosed in 83.6%, 16.3%, and 0.1% of patients, respectively. Notably, only 4% of individuals ≥120 kg received 3 g of cefazolin, thereby confounding the effects of underdosing and heavier body weight. The rate of PJI was higher in patients who were underdosed (1.51% versus 0.86%, *p* = 0.002) and in those ≥120 kg (3.25% versus 0.83%, *p* < 0.001). After multivariable analysis, only underdosing [odds ratio (OR) = 1.665, *p* = 0.006] and a higher Charlson comorbidity index (CCI) (OR = 1.259, *p* < 0.001) were independently associated with infection. The authors proposed that cefazolin underdosing, most evident in patients with obesity, contributed to a higher risk of PJI.

Morris et al. analyzed the relationship between cefazolin dose, body weight, and SSI in 38,288 cases of hip or knee arthroplasty, using data from a national (New Zealand) prospective, surveillance and quality improvement programme [28]. Underdosing was defined as 1 g for individuals ≥80 kg or <3 g for those ≥120 kg. The preoperative dose was administered within 1 h in 96% of cases, and surgery exceeded 4 h in only 1% of cases. The recommended cefazolin dose was given in 94.5% cases, noting that only 27% of patients ≥120 kg received 3 g. After multivariable analysis, underdosing (OR = 2.19, *p* < 0.0001), higher surgical risk score (*p* < 0.0001), revision (*p* < 0.0001) or hip surgery (*p* = 0.028), and male gender (*p* = 0.026) were independently associated with SSI. It was estimated that 83 heavier-weight individuals needed to be treated with the recommended dose to prevent one infection. The investigators suggested that 2 g and at least 3 g of cefazolin were appropriate for patients ≥80 kg and ≥120 kg, respectively, but were unable to address the potential need for higher doses in patients with morbid obesity undergoing arthroplasty.

Finally, in a retrospective, cohort study, Karamian et al. investigated whether inadequate cefazolin dosing affected infection rates in 2643 cases of spine surgery at a single center spanning 20 years [29]. An adequate dose was defined as 1 g for patients <60 kg, 2 g for those from 60 kg to 120 kg, and 3 g for those >120 kg. Again, only 5% of individuals >120 kg received 3 g of cefazolin. Whereas adequate dosing was independently associated with a lower risk of SSI (OR = 0.45, *p* < 0.001), higher BMI (*p* < 0.001), and higher CCI (*p* = 0.009) increased the risk of infection. In a subgroup analysis of cefazolin dosing, the 2 g dose was independently associated with a lower risk of SSI [OR = 0.20, *p* < 0.001], while heavier body weight increased the risk of infection [OR = 1.03, *p* < 0.001]. The relationship between body weight and SSI was studied further in a subset analysis using receiver operating characteristic (ROC) curves. Strong associations were observed between body weight and infection for both the 1 g and 2 g doses of cefazolin [area under the curve (AUC) = 0.85 (CI_95%_ 0.777 to 0.924) and AUC = 0.575 (CI_95%_ 0.493 to 0.657), respectively]. Body weight >80.75 kg was a significant threshold for cefazolin dosing, where using 1 g would increase the risk of infection by 85%. The authors proposed that 2 g of cefazolin was beneficial regardless of body weight but given the association between obesity and SSI, they were unable to confirm the need for 3 g in those >120 kg.

In conclusion, the findings of several large retrospective or observational studies support the importance of cefazolin dose for effective SAP. However, the correlation between heavier body weight and less mg/kg of cefazolin delivered by standard doses confounds the analysis of body weight and dose as risk factors for SSI. Such studies are also limited by relatively poor adherence to the recommended 3 g dose for patients ≥120 kg. Hence, high proportions of that group are concurrently labelled as underdosed in risk factor analyses. Even so, the SAP dose is a significant and modifiable risk factor that should be considered in situations like obesity, where altered PKs may reduce the antibiotic concentrations achieved in patients during surgery.

**Table 1:** Dose–response relationship in cefazolin prophylaxis: Studies of the association between lower cefazolin dose and SSI.

Study	Study Design	Surgery (Number of Patients, Years of Enrollment)	Key Findings Regarding Cefazolin Dose and SSI
Cies et al., 2012 [25]	Subgroup analysis of weight-based cefazolin dosing and MSSA SSI in pediatrics (mean 12.8 years, range 3–19 years), using data from a retrospective single-center, case–control study (n = 105 and 212 controls) of BMI as a risk factor for SSI within 30 days or 1 year if instrumentation placed. ^1^	Pediatric clean orthopedic surgery (n = 200, 2002–2005)	- SSI rate was higher with a standard dose of 1 g for patients ≥70 kg compared to a weight-based dose of 20–30 mg/kg to a maximum of 1 g for those <70 kg [35.9% vs. 20.5%, OR = 2.17 (CI_95_ 1.02–4.65), *p* = 0.045]; - SSI rate was higher in patients who were underweight compared to those who were a healthy weight (44.8% vs. 28.8%, *p* = 0.023); - In the overall cohort, ASA class II or III (*p* = 0.006 ) and surgery >2 h (OR = 2.45, *p* = 0.01) were independently associated with SSI. ^2^
Abdel Jalil et al., 2017 [26]	Prospective observational, single-center study of the frequency of SSI within 30 days and risk factors for infection including noncompliance with a new institutional guideline to use 2 g instead of 1 g of cefazolin preoperatively within 1 h for patients <120 kg.	Caesarean surgery (n = 861, 2015–2016)	- Calculated mg/kg dose of cefazolin received by patients was lower in patients with SSI compared to those without infection [18.7 mg/kg (CI_90%_ 10.6–28.6) vs. 22.2 mg/kg (CI_90%_ 11.1–29.9), *p* = 0.037]; - Lower mg/kg dose [OR = 0.976 (CI_95%_ 0.94–0.99), *p* = 0.035], longer hospital-stay (>3.5 days, *p* = 0.001), higher BMI (≥36 kg/m^2^, *p* = 0.003), and delivery beyond the 40th week (*p* = 0.005) were independently associated with SSI. ^2^
Rondon et al., 2018 [27]	Retrospective single-center study of cefazolin underdosing and the risk of PJI within 1 year, where underdosing was defined as cefazolin <1 g for any patient, <2 g for those between 60 kg and 120 kg, or <3 g for those ≥120 kg. ^3^	Total joint arthroplasty (n = 17,393, 2005–2017)	- Cefazolin was adequately dosed in 83.6%, underdosed in 16.3%, and overdosed in 0.1% of cases; 4% of patients >120 kg received 3 g; - PJI rate was higher in patients who were underdosed (1.51% vs. 0.86%, *p* = 0.002) and in those ≥120 kg (3.25% vs. 0.83%, *p* < 0.001); - Cefazolin underdosing (OR = 1.665, *p* = 0.006) and higher CCI (OR = 1.259, *p* < 0.001) were independently associated with PJI. ^2^
Morris et al., 2020 [28]	Retrospective study of the relationship between body weight, cefazolin dose, and SSI within 90 days, using data from a national (New Zealand), prospective surveillance and quality improvement programme, and where the recommended dose was cefazolin 1 g for patients ≥80 kg or <3 g for those ≥120 kg. ^4^	Knee or hip arthroplasty (n = 38,288, 2013–2017)	- Cefazolin was given at the recommended dose in 94.5% and underdosed in 5.5% of cases; 27% of patients >120 kg received 3 g; - SSI was higher in patients >120 kg (OR = 3.1, *p* < 0.001); - Cefazolin underdosing [OR = 2.19 (CI_95%_ 1.61–2.99), *p* < 0.0001], higher surgical risk score (*p* < 0.0001), revision (*p* < 0.0001) or hip (*p* = 0.028) surgery, and male gender (*p* = 0.026) were independently associated with SSI; ^2^ - Eighty-three (83) heavier-weight patients needed to be treated with the recommended dose to prevent one infection.
Karamian et al., 2022 [29]	Retrospective single-center cohort study of risk factors for SSI within 90 days, where adequate cefazolin dosing was defined as cefazolin 1 g for patients <60 kg, 2 g for those from 60 to 120 kg, and 3 g for those >120 kg. ^5^	Cervical or lumbar spinal fusion (n = 2643, 2000–2020)	- Cefazolin was inadequately dosed in 31% of cases; 5% of patients >120 kg received 3 g; - Adequate cefazolin dosing was independently associated with a lower risk of SSI [OR = 0.45 (CI_95%_ 0.30–0.69), *p* < 0.001], while a higher BMI (*p* < 0.001) and higher CCI (*p* = 0.009) increased the risk of infection;^2^ - In a subgroup analysis of cefazolin dosing, the 2 g dose was independently associated with a lower risk of SSI [OR = 0.20 (CI_95%_ 0.12–0.35), *p* < 0.001], while heavier body weight increased the risk of infection [OR = 1.03 (CI_95%_ 1.02–1.04), *p* < 0.001]. ^2^ For the 1 g dose, body weight >80.75 kg was a significant threshold for SSI. ^6^

ASA is American Society of Anesthesiologists, BMI is body mass index, CCI is Charlson comorbidity index, CI_95%_ 95% confidence interval, MSSA is methicillin-susceptible *Staphylococcus aureus*, OR is odds ratio, PJI is prosthetic joint infection, SSI is surgical site infection, vs. is versus. ^1^ Preincision dose within 1 h in 78.9% of cases; redosing during surgery needed in 6.9% of cases. ^2^ Multivariable logistic regression analysis. ^3^ Preincision dose within 1 h in all of cases; information on redosing during surgery and extended courses postsurgery not provided. ^4^ Preincision dose within 1 h in 96% of cases; surgery exceeded 4 h in 1% of cases. ^5^ Redosing during surgery in 31.1% of cases. ^6^ Receiver operating characteristic (ROC) curve analysis.

## 3. Antibiotic Concentration–Response (SSI) Relationship in SAP

In 1977, Goldmann et al. may have been the first to note an association between SSI and antibiotic concentrations in patients undergoing surgery [30]. They conducted a prospective, double-blinded trial of extended courses of cephalothin prophylaxis following prosthetic valve replacement. Although there was no benefit to prolonging the course of antibiotics, the investigators observed that patients without detectable cephalothin in their serum at the close of surgery were more likely to develop staphylococcal SSI [27.3% (3/11) versus 1.1% (2/175), *p* = 0.002]. A comprehensive literature search for the current review identified seven studies of antibiotic concentrations in serum/plasma or tissue collected during surgery and SSI in patients. As detailed in Table 2, there were two studies of cefazolin, two studies of ceftriaxone, and one study each of cefmetazole, cefuroxime, and gentamicin. Three of the studies measured antibiotic concentrations in serum/plasma, whereas four measured the amount of antibiotic in tissue (± serum/plasma concentrations).

The PKPD of gentamicin prophylaxis in colorectal surgery was studied as part of a prospective, double-blinded trial that compared a single-dose versus multiple-dose regimen of gentamicin and metronidazole (n = 134) [31,32]. In the PKPD analysis, several patient- and surgery-related variables were investigated as potential risk factors for SSI. Gentamicin preincision timing, serum concentrations at incision and closure, and AUC from incision to closure were also considered. After multivariable analysis, a lower gentamicin concentration at closure (*p* = 0.02), diabetes (*p* = 0.02), stoma (*p* = 0.04), and older age (*p* = 0.05) were independently associated with infection. Furthermore, a closure concentration <1.6 mg/L was a significant threshold for infection (ROC curve, *p* = 0.002, sensitivity = 70.8%, specificity = 65.9%). Other risk factors for SSI including earlier preincision timing and longer surgery were associated with lower closure concentration and not significant in multivariable analysis. Of note, the mg/kg dosing of gentamicin mitigated the association between heavier body weight and lower dose that confounded the study of cefazolin as previously discussed.

The PKPD of cefazolin prophylaxis was investigated as part of a prospective, non-interventional PK study of total and unbound cefazolin in patients undergoing cardiac surgery (n = 40) [33,34]. After multivariable analysis, a lower total plasma concentration at closure (OR = 1.3 per 10% decrease in concentration, *p* = 0.038) and longer surgery (OR = 2.9 per additional 1 h, *p* = 0.027,) were independently associated with SSI. In classification and regression tree (CART) analysis, a total cefazolin closure concentration <104 mg/L (~29 mg/L unbound) and surgery >346 min were significant thresholds for infection. Given the small sample size, the thresholds were more descriptive of the PKPD relationship than definitive targets. The stronger association with total as opposed to unbound cefazolin may have been due to significant variability in plasma albumin and protein binding during cardiac surgery.

Albacker et al. studied cefuroxime plasma concentrations and SSI in patients undergoing coronary artery bypass grafting (CABG) (n = 78) [35]. Concentrations before incision, before or 1 h after starting cardiopulmonary bypass (CPB), and before skin closure were not significantly different between patients with or without infection. Durations from the preoperative dose to starting CPB (*p* = 0.01) and to skin closure (*p* = 0.03) were longer in those who developed SSI. However, there was a notable infection rate of 18% during the study. The authors also reported that SAP was inappropriate in 78.2% of all cases including 42.3% with improper preincision timing and 44.9% with inadequate redosing. It is difficult to know how surgical prophylaxis practices at the time may have influenced the study findings.

Takayama et al. examined cefmetazole in serum and whole (fat) tissue during lower gastrointestinal surgery (n = 105) [36]. Subcutaneous tissue samples were collected at the time of incision and before skin closure. The tissue was pulverized in liquid nitrogen, homogenized in a phosphate buffer, and centrifuged to obtain supernatant for the analytical assay. In univariate analysis, a lower tissue concentration at closure (1.0 mg/g versus 2.2 mg/g, *p* = 0.09), not redosing during surgery (*p* = 0.09), lower creatinine clearance (*p* = 0.09), and older age (*p* = 0.02) were associated with SSI (α = 0.1). Although the amount of antibiotic in surgical tissue is important, the methodology and interpretation of such data must be carefully considered [37,38]. Antibiotic tissue concentrations in the extracellular space, where bacteria contaminate the wound, are most relevant. Since only free antibiotic diffuses between the plasma and interstitial fluid, unbound plasma concentrations are more indicative of the pharmacologically active antibiotic in extracellular tissue. Microdialysis studies can be used to measure antibiotic in interstitial fluid that is collected from semipermeable catheters inserted or surgically placed in the tissue of interest. The probes are flushed with perfusion fluid, thereby allowing interstitial fluid to be continuously sampled during surgery. In contrast, whole tissue studies collect samples at specific times and measure the overall average amount of antibiotic per volume/weight of tissue. Since the methods lack standardization and procedures for quality control, the results are difficult to interpret [37,38]. Most techniques involve homogenizing whole tissue into a suspension of its cellular, extracellular, and vascular components, and then processing the sample to obtain supernatant. Noting that interstitial fluid accounts for only 10% to 30% of tissue volume, whole tissue concentrations do not reflect the antibiotic’s distribution or pharmacological activity in vivo. Since β-lactams like cefmetazole distribute into interstitial fluid, the concentrations in pulverized adipose tissue are diluted, thereby underestimating those in extracellular tissue [39]. Despite the limitations, however, the findings of Takamaya et al. support an antibiotic concentration–response relationship for cefmetazole prophylaxis [36]. The tissue concentrations at closure, using consistent sampling and analytical methods, were significantly lower in patients who developed SSI. Although it signals the importance of maintaining antibiotic in tissue during surgery, the specific concentrations are not clinically applicable and can not be compared to MICs or otherwise used to predict antibacterial activity at the surgical site.

Sheikh et al. investigated ceftriaxone in serum and whole (fat) tissue during pediatric clean or clean-contaminated surgery (n = 50) [40]. The mean age of patients was 6.1 years, ranging from 2 years to 12 years. The samples were collected at the time of incision, midway, and at wound closure. The tissue was crushed, homogenized by sonification, reconstituted with normal saline, and centrifuged to obtain supernatant. Only longer surgery (*p* = 0.02), higher wound classification (*p* = 0.035), urinary catheter (*p* = 0.029), and implantation were associated with SSI (*p* = 0.002). The investigators also noted that ceftriaxone serum concentrations during surgery were always at least 20 times their threshold of 4 mg/L. Given the pediatric dosing (i.e., 75 mg/kg to a maximum of 1 g), it is possible that ceftriaxone concentrations exceeded the PKPD window where the relationship with SSI would be observed. Sheikh et al. also published a similar study of ceftriaxone in serum and whole (fat) tissue during adult spinal surgery (n = 50) [41]. Patients received a standard 1 g dose of ceftriaxone preoperatively. The same sampling, processing, and analytical methods were used. In this case, lower serum (*p* = 0.003) and tissue (*p* = 0.008) concentrations at closure, longer surgery (*p* = 0.003), and implantation (*p* = 0.049) were significantly associated with SSI in adults undergoing spinal surgery.

Finally, Byers et al. conducted a retrospective study of SAP in megaprosthetic reconstruction to determine whether extended courses of prophylaxis reduced the risk of PJI [42]. The study also included a PK subgroup analysis of cefazolin concentrations in surgical tissues of five patients who developed infection and five procedure-matched controls. Samples were obtained from punch biopsies of cortical bone and adjacent skeletal muscle collected during surgery. Muscle tissue was selected because of its high vascularization compared to other soft tissue, e.g., subcutaneous adipose. After the sample was cryo-crushed under liquid nitrogen, a novel multiple-step process was used to extract cefazolin from the paraffin-embedded tissue. For the primary aim of the study, the investigators found no benefit to prolonging antibiotics following surgery. In the PK analysis, however, cefazolin concentrations during surgery in bone (0.065 ng/mL versus 0.42 ng/mL, *p* < 0.01) and skeletal muscle (0.2 ng/mL versus 1.95 ng/mL, *p* = 0.03) were significantly lower in patients who developed PJI compared to controls. The authors suggested that, as opposed to extending the duration, opportunities to improve SAP in megaprosthetic reconstruction may reside in selecting the best antibiotic and ensuring adequate tissue concentrations during surgery.

In summary, available evidence supports an association between lower antibiotic concentrations in patients undergoing surgery, particularly at wound closure, and an increased risk of infection. However, the number of studies is relatively small. More research is needed to fully understand the principles of antimicrobial PKPD in surgical prophylaxis and realize its potential to improve SSI outcomes. Further study of antibiotic concentrations in serum/plasma and interstitial fluid during surgery can also help identify clinically meaningful targets for effective SAP.

**Table 2:** Studies of an antibiotic concentration–response (SSI) relationship in SAP.

Study	Study Design	Antibiotic ^1^	Surgery	Measured Antibiotic Concs (Analytical Assay)	Number of SSI Cases and Comparators or Controls ^2^	Key Findings Regarding Antibiotic Concentration and SSI
Zelenitsky et al., 2002 [31]	PKPD subgroup analysis in a prospective double-blinded trial of a single-dose vs. multiple-dose regimen of gentamicin and metronidazole prophylaxis (n = 146) [32]. ^3^	Gentamicin	Colorectal surgery	Total serum concs at least 30 min after the preoperative dose and postsurgery in recovery (fluorescence immunoassay)	33 superficial SSIs and 10 deep SSI within 30 days vs. 91 without infection	- Lower serum conc at closure (*p* = 0.02), diabetes (*p* = 0.02), stoma (*p* = 0.04), and older age (*p* = 0.05) were independently associated with SSI; ^4^ - Serum conc at closure <1.6 mg/L was a significant threshold for SSI (*p* = 0.002, sensitivity = 70.8%, specificity = 65.9%). ^5^
Zelenitsky et al., 2018 [33]	PKPD subgroup analysis in a prospective non-interventional PK study of cefazolin prophylaxis (n = 55) [34]. ^6^	Cefazolin	CABG, cardiac valve repair or replacement ^7^	Total and unbound plasma concs 30 min after the preoperative dose, prior to redosing, and within 15 min of wound closure (HPLC-tandem mass spectrometry)	8 superficial SSIs within 30 days vs. 32 without infection	- Lower total plasma conc at closure (OR = 1.3 per 10% decrease, *p* = 0.038) and longer surgery (OR = 2.9 per 1 h increase, *p* = 0.027) were independently associated with SSI; ^4^ - Total plasma conc at closure <104 mg/L and surgery >346 min were significant thresholds for SSI. ^8^
Albacker et al., 2022 [35]	Prospective PK study of cefuroxime prophylaxis. ^9^	Cefuroxime	CABG ^7^	Total serum concs immediately before incision, before and 1 h after starting CPB, and before skin closure (analytical assay not described)	14 SSIs (superficial within 30 days and deep/organ space within 90 days) vs. 64 without infection	- Serum concs were not different in patients with SSI compared to those without infection; - Longer durations from the preoperative dose to starting CPB (*p* = 0.01) and to skin closure (*p* = 0.03) were associated with SSI. ^10^
Takayama et al., 2022 [36]	Prospective PK study of cefmetazole prophylaxis. ^11^	Cefmetazole	Colectomy (laparoscopic), proctectomy	Total serum concs at the time of incision, intestinal resection, redosing, and skin closure, and whole adipose tissue concs at the time of incision and skin closure (HPLC)	7 superficial SSIs, 2 deep SSIs, and 4 organ space SSIs within 30 days vs. 92 without infection	- Lower tissue conc at closure (1.0 vs. 2.2 mg/g, *p* = 0.09), not redosing during surgery (*p* = 0.09), lower creatinine clearance (*p* = 0.09), and older age (*p* = 0.02) were associated with SSI (α = 0.1). ^10^
Sheikh et al., 2022 [40]	Prospective PK study of ceftriaxone prophylaxis in pediatrics (mean 6.1 years, range 2–12 years). ^12^	Ceftriaxone	Pediatric clean or clean-contaminated surgery (multiple)	Total serum and whole adipose tissue concs simultaneously at the time of incision, mid surgery, and wound closure (HPLC)	3 SSIs vs. 47 without infection (surveillance period not described)	- Lower tissue concs at closure (*p* = 0.04) but higher tissue concs at incision (*p* = 0.02) were correlated with SSI; - Longer surgery (*p* = 0.02), higher wound classification (*p* = 0.035), urinary catheter (*p* = 0.029), and implantation were associated with SSI (*p* = 0.002). ^13^
Sheikh et al., 2023 [41]	Prospective PK study of ceftriaxone prophylaxis. ^14^	Ceftriaxone	Spinal surgery	Total serum and whole adipose tissue concs simultaneously at the time of incision, mid surgery, and wound closure (HPLC)	4 SSIs vs. 46 without SSI (surveillance period not described)	- Lower serum conc at closure (64.25 vs. 84.46 mg/L, *p* = 0.003) and lower tissue conc at closure (6.90 vs. 8.82 mg/L, *p* = 0.008) were correlated with SSI; - Lower serum (*p* = 0.003) and tissue (*p* = 0.008) concs at closure, longer surgery (*p* = 0.003), and implantation (*p* = 0.049) were associated with SSI. ^13^
Byers et al., 2022 [42]	PK subgroup analysis in a retrospective study of extended courses of antimicrobial prophylaxis postsurgery (n = 184). ^15^	Cefazolin	Megaprosthetic reconstruction	Whole tissue concs in cortical bone and adjacent skeletal muscle from punch biopsies collected during surgery (HPLC-tandem mass spectrometry)	5 PJIs within 1 year vs. 5 procedure-matched controls without infection	- Tissue concs during surgery in cortical bone (0.065 vs. 0.42 ng/mL, *p* < 0.01) and adjacent skeletal muscle (0.20 vs. 1.95 ng/mL, *p* = 0.03) were lower in patients with PJI compared to controls.

CABG is coronary artery bypass grafting, Conc is concentration, CPB is cardiopulmonary bypass, HPLC is high-performance liquid chromatography, OR is odds ratio, PJI is prosthetic joint infection, PK is pharmacokinetic, PD is pharmacodynamic, SAP is surgical antimicrobial prophylaxis, SSI is surgical site infection, vs. is versus. ^1^ Intravenous administration. ^2^ Number of cases included in the analysis of a concentration–response relationship. ^3^ Serum creatinine <150 umol/L for inclusion. Single-dose regimen of gentamicin 4.5 mg/kg and metronidazole 500 mg preoperatively compared to multiple-dose regimen of gentamicin 1.5 mg/kg and metronidazole 500 mg preoperatively and at 8, 16, and 24 h postsurgery. ^4^ Multivariable logistic regression analysis. ^5^ Receiver operating characteristic (ROC) curve. ^6^ Creatinine clearance ≥50 mL/min for inclusion. Cefazolin by protocol, i.e., 1–2 g preoperatively within 1 h, repeated every 4 h during surgery, and every 8 h for 2 days postsurgery. ^7^ Surgery involving cardiopulmonary bypass. ^8^ Classification and regression tree (CART) analysis. ^9^ Cefuroxime dosing described as “according to the Society of Thoracic Surgeons Practice Guideline Series”. ^10^ Univariate analysis,. ^11^ Cefmetazole by protocol, i.e., 1–2 g preoperatively within 1 h, repeated every 3 h during surgery. ^12^ Ceftriaxone by protocol, i.e., 75 mg/kg preoperatively to a maximum of 1 g after induction. ^13^ Correlation test with ceftriaxone serum and tissue concentration and patient characteristics as independent variables, and SSI as the dependent variable. Binary logistic regression analysis of associations between SSI with each individual predictable variable and various concentrations. ^14^ Ceftriaxone by protocol, i.e., 1 g preoperatively after induction. ^15^ Cefazolin by protocol, i.e., 1–3 g preoperatively within 1 h, repeated every 4 h during surgery, and for a median of 3 days postsurgery.

## 4. Relevance and Role of PKPD in SAP

### 4.1. Antimicrobial PKPD and Monte Carlo Simulations

Antimicrobial PKPD is founded on decades of research in in vitro models, animal models, and patients [10,12,43,44]. Unlike medications with a direct therapeutic effect in patients, antibiotics work through their activity against an infecting pathogen. Therefore, antimicrobial PKPD incorporates the susceptibility of the isolate, represented by the MIC or lowest antibiotic concentration required to inhibit visible growth of the isolate during in vitro incubation [45]. Three PKPD indices of clinical outcome have been established for the treatment of infections. They include the maximum unbound [i.e., free (ƒ)] concentration divided by the MIC (ƒC_max_/MIC), the percentage of the dosing interval that unbound concentrations exceed the MIC (ƒ%T_>MIC_), and the unbound AUC over 24 h divided by the MIC (ƒAUC/MIC). ƒC_max_/MIC is most relevant for antibiotics with concentration-dependent activity, e.g., aminoglycosides while ƒ%T_>MIC_ applies to those with time-dependent effects, e.g., β-lactams. ƒAUC/MIC is a blended index of overall exposure for antibiotics like the fluoroquinolones and vancomycin. Although minimum therapeutic targets have been established such as a ƒC_max_/MIC > 8–10 for aminoglycosides, %ƒT_>MIC_ > 50% for cephalosporins, and AUC/MIC of 400 to 600 for vancomycin, higher thresholds may be warranted, e.g., serious infections, critical illness [12,46,47].

Monto Carlo simulation studies are important in antimicrobial PKPD research and knowledge translation [12,48]. They can integrate the complexity of numerous and often interdependent variables related to the patient, antibiotic, and infection. And they can incorporate real-world variability into patient characteristics (e.g., age, body weight, renal function) and PK parameters (e.g., Vd, t½, protein binding). Instead of interpolating based on an average or typical case, simulations can predict the probability of outcomes for an infinite number of “what-if-scenarios”. Monte Carlo simulations are increasingly used to evaluate and compare SAP regimens. First, the surgical population is generated according to the desired patient characteristics. The SAP is replicated in each subject, and antibiotic concentration curves are generated based on PK models. The use of relevant, high-quality PK data is essential to produce reliable and translatable simulation results. Ideally, population PK models that describe covariate relationships between parameters, such as body weight and Vd, or renal function and t½, are used [49]. Unbound concentrations may be simulated using the percentage of protein binding or population PK data for unbound drug if available. The concentration curves of each subject are considered against the most likely SSI pathogens, and the probability of achieving the desired PKPD target is predicted for the population. Since the goal of SAP is to maintain adequate antibiotic concentrations throughout surgery, the relevant PKPD target is to achieve 100%ƒT_>MIC_, from incision to closure. Unlike the treatment of infections, however, the MIC is speculative, and the concentration threshold used to evaluate SAP is selected by the investigator. As such, Monte Carlo simulations are typically designed to determine the probability of target attainment (PTA) for 100%ƒT_>MIC_ using a specific MIC believed to be the lowest acceptable concentration for surgical prophylaxis or the fractional target attainment (FTA) using the distribution of MICs for a population of bacteria. In all cases, simulation studies must be considered carefully with regards to their methods, input data, analysis, and interpretation of results.

### 4.2. PKPD Considerations for Improving Cefazolin Prophylaxis

SAP is supported by several well-established practice guidelines and recommendations for the prevention of SSI [8,20,21,22]. Although there is consensus that SAP should provide adequate coverage throughout surgery, there are questions regarding the best approaches. A recent review by an international panel of experts identified “six long-standing questions about antibiotic prophylaxis in surgery” including: How should the dose be chosen, when should it be administered, and when should it be redosed intraoperatively [50]? Those questions are explored from a PKPD perspective, with a focus on optimizing the dose, preincision timing, and redosing of cefazolin prophylaxis in adults. Although the concepts may apply to other antibiotics, the applications and discussions that follow are specific to cefazolin.

#### 4.2.1. Pharmacokinetics of Cefazolin Prophylaxis

Cefazolin is a small hydrophilic compound with PKs characterized by an average protein binding of 80%, apparent Vd of 0.15 L/kg, and terminal t½ with normal renal function of 2 h [51]. The PKs of cefazolin are variable in patients depending on factors such as age, sex, body weight and composition, renal function, and comorbidities. The concentration curve for a bolus dose of cefazolin can be described by a two-compartment model, where the central compartment represents intravascular space (i.e., serum/plasma), and the peripheral compartment signifies extracellular tissue (i.e., interstitial fluid). The C_max_ in plasma is determined by the dose, infusion, and central Vd. The transiently high plasma concentrations following a bolus dose are less clinically relevant since free cefazolin rapidly diffuses across the concentration gradient into tissue. The post-distributional concentrations decline during the elimination phase primarily due to renal clearance. The tissue penetration of antibiotics is an important consideration in the treatment and prevention of infections [38]. The diffusion of an antibiotic into tissue depends on its physiochemical properties, favoring those with smaller size, higher lipophilicity, and lower protein binding. The tissue characteristics are also relevant where well vascularized sites without additional barriers are more accessible. Hence, there is lower cefazolin penetration into less perfused surgical sites like bone [52,53] and, in particular, excess adipose tissue. Finally, the rate and extent of tissue penetration is variable, as demonstrated by the study of cefazolin concentrations in the adipose tissue of patients undergoing surgery [54,55,56,57,58].

Standardized protocols are often used to facilitate SAP in clinical setting, e.g., 2 g of cefazolin for patients <120 kg or 3 g for those ≥120 kg administered within 1 h prior to incision and repeated every 2 t½’s during surgery [8]. As expected, cefazolin prophylaxis produces a wide range of concentration curves where, for example, 2 g may be given to patients who weigh up to 120 kg and have any degree of renal function. Given the limited number of SAP doses, there is less concern about cefazolin overdosing and accumulation. Other the other hand, it is important to identify when altered PKs may lower antibiotic concentrations during surgery, thereby increasing the risk of SSI, e.g., neonates, obesity, pregnancy, cardiac surgery.

#### 4.2.2. Cefazolin Prophylaxis Dose and Obesity

Although there is broad support for the 2 g dose of cefazolin for patients <120 kg [27,29], the 3 g dose for those ≥120 kg is controversial. In 2022, Coates et al. published a systematic review of cefazolin prophylaxis and dosing in patients with obesity [59]. Three SSI outcome studies were identified including one in caesarean surgery where patients ≥131.5 kg received either 2 g or 3 g of cefazolin preoperatively [60]. Two studies were conducted in elective surgery where one compared 2 g to 3 g in patients ≥100 kg [61], and the other examined 2 g in those <120 kg and ≥120 kg [62]. None of the outcome studies supported the higher dose of cefazolin for patients with obesity. Fifteen PK studies that evaluated serum/plasma or tissue concentrations against an MIC-based target of 100%ƒT_>MIC_ were also reviewed. Nine studies concluded that 2 g of cefazolin was sufficient for patients with obesity, whereas six found that it was insufficient. As noted in the review, the PK studies were difficult to interpret given the variability in patients, surgeries, sampling sites, and PK modelling and simulation methods. Most notably, the MIC threshold used to determine %ƒT_>MIC_ and evaluate cefazolin doses ranged from 1 mg/L to 8 mg, limiting the ability to compare study results. Without evidence to the contrary, Coates et al. proposed that 2 g of cefazolin was sufficient for patients with obesity undergoing surgery lasting up to 4 h.

Subsequently, Ryan et al. published a population PK study of unbound cefazolin in the plasma and interstitial fluid of subcutaneous abdominal tissue (microdialysis) during elective bariatric surgery [58]. Patients with body weights ranging from 99 kg to 198 kg (median 154 kg) received 2 g of cefazolin preoperatively. The PKs of unbound cefazolin were described by a four-compartment model with nonlinear protein binding and a covariate relationship between BMI and Vd. In Monte Carlo simulations, at least 95% of simulated subjects achieved a concentration threshold of >2 mg/L in interstitial fluid throughout surgery. The authors concluded that 2 g of cefazolin given 30 min to 60 min preincision was adequate for patients with obesity or morbid obesity undergoing bariatric surgery lasting up to 4 h (FTA 97.8% to 98.5%).

**Figure 1:**
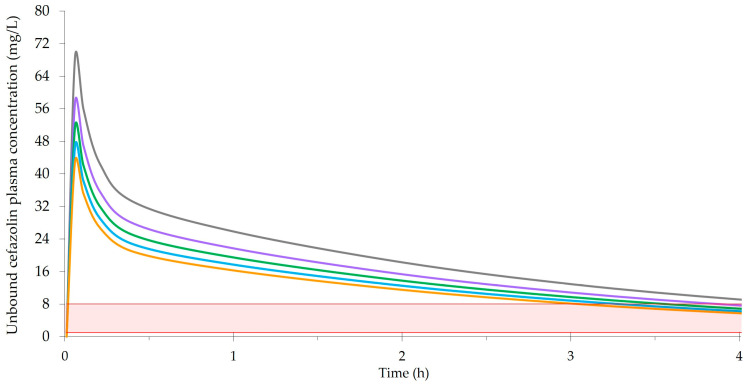
Illustration of concentrations following a 2 g bolus dose of cefazolin prophylaxis, where the grey line represents 60 kg (BMI 21), the purple line represents 90 kg (BMI 31), the green line represents 120 kg (BMI 42), the blue line represents 150 kg (BMI 52), and the orange line represents 180 kg (BMI 62). The shaded area is the range of investigator-selected MIC thresholds (i.e., 1 mg/L to 8 mg/L) used to determine %ƒT_>MIC_ and evaluate cefazolin doses in the PK studies included in the systematic review by Coates et al. [59].

Figure 1 exemplifies a two-compartment model of unbound plasma concentrations following 2 g of cefazolin preoperatively for different body weights. It was created based on a population PK study of total and unbound cefazolin in major surgery by Naik et al. [63]. Their PK model was characterized by a median clearance of 4.65 L/h, central volume (Vc) of 5.63 L, distribution rate constant from the central to peripheral compartment of 4.88 h^−1^, distribution rate constant from the peripheral to centration compartment of 3.98 h^−1^, and a covariate relationship between body weight and volume where Vc = Vc (typical) × (body weight/80) ^0.75^. Figure 1 uses data from Naik et al. to estimate Vd. However, since cefazolin penetrates poorly into adipose tissue, the Vd for body weights ≥ 120 kg incorporates an adjusted dosing weight (adjDW) where adjDW = ideal body weight + [0.4 × (total body weight − ideal body weight)] [64]. Figure 1 also uses an average protein binding of 21% [63] and terminal t½ of 2 h, representing normal renal function and an elimination unaffected by obesity [56,65,66].

PK studies can show relatively small differences in cefazolin concentrations with the same dose for heavier body weights extending into obesity and morbid obesity. This is explained by the distribution of cefazolin, and most β-lactams, into extracellular tissue which lessens the effects of obesity on Vd. As shown in Figure 1, the differences with 2 g of cefazolin for body weights ranging from 60 kg to 180 kg are relatively modest and narrow over time. Most relevant to surgical prophylaxis, the cefazolin concentrations are similar at a redosing time of 3 h to 4 h. However, cefazolin concentrations in interstitial fluid, represented by unbound plasma concentrations as in Figure 1 or measured using microdialysis, must be considered in context. Given the excess adipose tissue, there is significantly less interstitial fluid in the tissue of patients with obesity. Since adipose tissue is also poorly vascularized, the amount of antibiotic available at the surgical site is further reduced. Therefore, a cefazolin dose (e.g., 2 g) that produces similar cefazolin concentrations in the interstitial fluid of nonobese and obese patients does not necessarily provide comparable surgical prophylaxis. Given the lower amounts of interstitial fluid and blood flow in adipose tissue, it is possible that higher interstitial fluid concentrations and PKPD targets would be beneficial in obesity. It is an important consideration that supports using a higher cefazolin dose for heavier body weights. Although strong evidence for the 3 g dose in patients ≥120 kg is lacking, as previously discussed, clinical studies of cefazolin dose and SSI have limitations that make it difficult to detect differences that may exist between doses. Furthermore, PK-simulation studies may not consider the potential need for higher concentrations in obesity. Although the weight-based cut-offs of 120 kg and 35 kg/m^2^ have been shown to poorly predict those who required more than 2 g of cefazolin [66], better approaches are not available at this time. Finally, whereas the additional risks of using a 3 g versus 2 g dose are minimal, the benefits include increasing cefazolin concentrations in interstitial fluid, extending the duration of prophylaxis coverage, and potentially preventing SSIs in a high-risk patient population.

#### 4.2.3. Preincision Timing of Cefazolin Prophylaxis

SAP must be administered prior to incision to be effective [67,68,69]. Although there is general agreement to give the first dose within 60 min of incision, a 120 min window may be acceptable when considering antibiotics with long t½’s or prolonged infusions, e.g., vancomycin [21]. There are also suggestions of added benefit for specific times within the 60 min window. While some studies reported lower infection rates when SAP was administered closer to incision [68,70,71,72], others found no difference [73,74,75] or a higher rate of SSI [76]. In 2017, de Jong et al. published a meta-analysis of different SAP timing intervals for 54,552 patients from 14 studies [69]. Although the risk of SSI was significantly higher when SAP was given more than 120 min before incision (OR = 5.26, CI_95%_ 3.29–8.39) or after incision (OR = 1.89, CI_95%_ 1.05–3.40), differences were not observed for specific times within the 120 min window. The authors also noted considerable heterogeneity in the antibiotics and dosing regimens, and the evidence overall was low to moderate.

**Figure 2:**
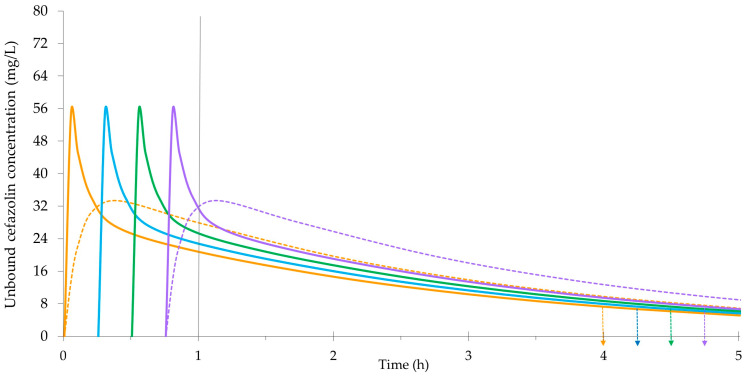
Illustration of concentrations following a 2 g bolus dose of cefazolin prophylaxis (80 kg), where incision is at 1 h, and the orange lines represent a timing 60 min prior to incision (solid line is plasma, hatched line is interstitial fluid), the blue line represents a timing 45 min prior to incision, the green line represents a timing 30 min prior to incision, and the purple lines represent a timing 15 min prior to incision (solid line is plasma, hatched line is interstitial fluid). The arrows represent the respective times for redosing at 4 h.

Figure 2 exemplifies the potential effects of preincision timing on a 2 g bolus dose of cefazolin for a body weight of 80 kg. It is based on the same population-PK model and data described for Figure 1 [63]. In addition to unbound plasma concentrations, Figure 2 displays cefazolin concentrations in interstitial fluid. The tissue concentrations increase more gradually as free cefazolin diffuses into the peripheral compartment. The rate of tissue penetration can also be characterized by the time to achieve maximum cefazolin concentrations (T_max_) following a preoperative dose. One study in abdominal aortic aneurysm repair surgery observed rapid diffusion into subcutaneous tissue (microdialysis) with a median T_max_ of 2.0 min [54]. Separate investigations of cefazolin penetration into the skeletal muscle (microdialysis) of pediatric patients reported a median T_max_ of 37.6 min in cardiac surgery, and values from 30 min to 38.5 min in spinal surgery [77,78]. The findings of such studies are variable and significantly influenced by the methods for sampling and data analysis. As depicted by the concentration curves in Figure 2, adjusting the preincision timing to ensure maximum tissue concentrations at the time of incision is unlikely to be beneficial or necessary.

#### 4.2.4. Redosing Cefazolin Prophylaxis during Surgery

Redosing during surgery may be required to ensure adequate cefazolin concentrations at closure [79]. In 2022, Wolfhagen et al. published a meta-analysis of SAP redosing for 9470 patients from 10 studies [80]. Redosing was associated with a significant reduction in SSI in the observational cohort studies only (OR = 0.55, CI_95%_ 0.38–0.79). Again, there was notable heterogeneity in the antibiotics and redosing protocols, leading to an overall low certainty of the evidence. Based on the longstanding recommendation of redosing every 2 t½’s, cefazolin is typically given every 3 h or 4 h assuming normal renal function. As kidney function declines, the duration of cefazolin prophylaxis coverage is extended and redosing may not be required [81]. On the other hand, more aggressive redosing may be used in situations where standard SAP regimens are inadequate. For example, cefazolin redosing after 2 h was suggested for patients >35 kg/m^2^ undergoing cesarean surgery [82]. In cardiac surgery with CPB, cefazolin redosing every 3 h was recommended for patients with normal renal function [34], whereas some described redosing every 2 h [83]. Other strategies to augment cefazolin prophylaxis in cardiac surgery include additional doses after starting CPB [84] or at skin closure [85]. Cefazolin prophylaxis given as a bolus dose preoperatively followed by continuous infusion during surgery eliminates the need for redosing and may also lower the risk of SSI in cardiac surgery [83,86].

#### 4.2.5. Conclusions

Optimal antibiotic dosing is based on the principles of antimicrobial PKPD. For SAP, PKPD relates antibiotic exposure during surgery to the subsequent development of SSI. It can incorporate the complexity of numerous and often interdependent variables related to the patient, antibiotic regimen, concentrations achieved in patients, and susceptibilities of the most likely SSI pathogens. It also shows the inextricable connection between SAP dose, preincision timing, and redosing, and why it is so difficult to isolate their effect on SSI outcomes in clinical studies.

## 5. Materials and Methods

A comprehensive literature search was conducted on the antibiotic concentration–response (SSI) relationship in SAP. The search aimed to identify articles with (1) measured antibiotic concentrations in serum/plasma or tissue samples collected during surgery and (2) surveillance for SSI. Since a relatively small number of eligible studies was expected, a broad search strategy was employed. Keyword and subject heading searches were conducted from inception to September 2023 in Ovid MEDLINE and Embase on the concepts of antibiotic (antimicrobial) prophylaxis, surgical site (wound) infection, pharmacokinetics, and pharmacodynamics. Citation chaining in Google Scholar and Scopus was carried out to find additional articles. Studies in animals and languages other than English were excluded, as were studies with simulated antibiotic concentrations only or SSIs that were <5% of cases analyzed for a concentration–response relationship. The titles and abstracts of all articles were screened and, if needed, full texts were reviewed to determine relevance. Finally, the reference lists of eligible studies were examined to identify articles that may have been missed.

## Abbreviations

adjDW	Adjusted dosing weight
ASA	American Society of Anesthesiologists score
AUC	Area under the curve
AUC/MIC	AUC over 24 h divided by the minimum inhibitory concentration
BMI	Body mass index
CABG	Coronary artery bypass grafting
CART	Classification and regression tree (analysis)
CCI	Charlson comorbidity index
CI	Confidence interval
C_max_	Maximum concentration
C_max_/MIC	Maximum concentration divided by the minimum inhibitory concentration
CPB	Cardiopulmonary bypass
CTA	Cumulative target attainment
ƒ	Free or unbound (i.e., not bound to protein)
FTA	Fractional target attainment
HPLC	High-performance liquid chromatography
MIC	Minimum inhibitory concentration
MSSA	Methicillin-susceptible *Staphylococcus aureus*
OR	Odds ratio
PD	Pharmacodynamic
PJI	Prosthetic joint infection
PK	Pharmacokinetic
PKPD	Pharmacokinetic-pharmacodynamic
ROC	Receiver operating characteristic (curve)
SAP	Surgical antimicrobial prophylaxis
%T_>MIC_	Percentage of the dosing interval that concentrations exceed the minimum inhibitory concentration
T_max_	Time to maximum concentration
t½	Half-life
Vd	Volume of distribution
